# Mutational analysis of the spike protein of SARS-COV-2 isolates revealed atomistic features responsible for higher binding and infectivity

**DOI:** 10.3389/fcell.2022.940863

**Published:** 2023-01-17

**Authors:** Muhammad Hanifa, Muhammad Salman, Muqaddas Fatima, Naila Mukhtar, Fahad N. Almajhdi, Nasib Zaman, Muhammad Suleman, Syed Shujait Ali, Yasir Waheed, Abbas Khan

**Affiliations:** ^1^ Centre for Biotechnology and Microbiology, University of Swat, Charbagh, Khyber Pakhtunkhwa, Pakistan; ^2^ Rashid Latif Medical College, Lahore, Punjab, Pakistan; ^3^ King Edward Medical University, Lahore, Punjab, Pakistan; ^4^ Department of Botany, University of Okara, Punjab, Pakistan; ^5^ COVID-19 Virus Research Chair, Department of Botany and Microbiology, College of Science, King Saud University, Riyadh, Saudi Arabia; ^6^ Office of Research, Innovation and Commercialization, Shaheed Zulfiqar Ali Bhutto Medical University (SZABMU), Islamabad, Pakistan; ^7^ Gilbert and Rose-Marie Chagoury School of Medicine, Lebanese American University, Byblos, Lebanon; ^8^ Department of Bioinformatics and Biological Statistics, School of Life Sciences and Biotechnology, Shanghai Jiao Tong University, Shanghai, China

**Keywords:** SARS-CoV-2, variants, genomes, spike protein, docking, simulation

## Abstract

**Introduction:** The perpetual appearance of Severe Acute Respiratory Syndrome Coronavirus 2 (SARS-COV-2), and its new variants devastated the public health and social fabric around the world. Understanding the genomic patterns and connecting them to phenotypic attributes is of great interest to devise a treatment strategy to control this pandemic.

**Materials and Methods:** In this regard, computational methods to understand the evolution, dynamics and mutational spectrum of SARS-CoV-2 and its new variants are significantly important. Thus, herein, we used computational methods to screen the genomes of SARS-CoV-2 isolated from Pakistan and connect them to the phenotypic attributes of spike protein; we used stability-function correlation methods, protein-protein docking, and molecular dynamics simulation.

**Results:** Using the Global initiative on sharing all influenza data (GISAID) a total of 21 unique mutations were identified, among which five were reported as stabilizing while 16 were destabilizing revealed through mCSM, DynaMut 2.0, and I-Mutant servers. Protein-protein docking with Angiotensin-converting enzyme 2 (ACE2) and monoclonal antibody (4A8) revealed that mutation G446V in the receptor-binding domain; R102S and G181V in the N-terminal domain (NTD) significantly affected the binding and thus increased the infectivity. The interaction pattern also revealed significant variations in the hydrogen bonding, salt bridges and non-bonded contact networks. The structural-dynamic features of these mutations revealed the global dynamic trend and the finding energy calculation further established that the G446V mutation increases the binding affinity towards ACE2 while R102S and G181V help in evading the host immune response. The other mutations reported supplement these processes indirectly. The binding free energy results revealed that wild type-RBD has a TBE of −60.55 kcal/mol while G446V-RBD reported a TBE of −73.49 kcal/mol. On the other hand, wild type-NTD reported −67.77 kcal/mol of TBE, R102S-NTD reported −51.25 kcal/mol of TBE while G181V-NTD reported a TBE of −63.68 kcal/mol.

**Conclusions:** In conclusion, the current findings revealed basis for higher infectivity and immune evasion associated with the aforementioned mutations and structure-based drug discovery against such variants.

## 1 Introduction

Coronavirus disease (COVID-19), caused by a new beta coronavirus called severe acute respiratory syndrome coronavirus 2 (SARS-CoV-2), was declared a pandemic by the World Health Organization (WHO) on 11 March 2020 (Isabel et al., 2020). After emerging in Wuhan, China, at the end of December, SARS-COV-2 spread to over 200 countries by mid-February (Zumla et al., 2016; Gobeil et al., 2021). Respiratory transmission is the main route through which the virus passes from one host to another. The novel coronavirus 2019 genome encodes four structural proteins (S, M, E, and N), in which S protein gives the virus its corona-like shape that is mainly responsible for the attachment to the host cell receptor (ACE2) or surface protein and 16 non-structural proteins (nsp-1 to nsp-16). The binding of the spike protein to ACE2 of the host initiates the infection in cells. ACE2 is mainly expressed in the lungs, kidney, and small intestine, leading to serious illness (Duchene et al., 2020). During infection, the host cell protease cleaves the S protein at S1/S2 cleavage site. This priming (cleavage of S protein) results in the division of protein into S1-ectodomain at N-terminal and S2 membrane-anchored domain at C-terminal. The S1 subunit recognizes the associated cell surface receptor, while the latter assists the viral entry (Belouzard et al., 2012; Tortorici and Veesler, 2019; Yan et al., 2020). The SARS-CoV-2 S1 subunit of the spike protein has conserved 14aa in the receptor-binding domain (RBD), which functions to recognize ACE2 and can infect both humans and bats. Among this conserved 14aa in SARS-CoV-2, eight residues are highly conserved in novel coronavirus 2019 (2019-nCoV), supporting the assumption that ACE2 is also a receptor of this new virus (Li et al., 2003).

Genome sequencing insights have shown the nucleotide substitution rate as ∼1 × 10^–3^ per year for SARS-CoV-2 (Duchene et al., 2020; Hussain et al., 2020). Variations in different proteins of SARS-CoV-2, particularly spike glycoprotein, lead to a drift in the antigenicity of vaccines or other therapeutics (Peacock et al., 2021a). Single amino acid substitution in the protein sequence results in structural changes, affecting a protein’s function. The substitution of D614 with G614 in spike glycoprotein causes changes in the conformation of cleavage site loop, leading to more effective S1 and S2 cleavages by enhancing furin accessibility (Peacock et al., 2021b). As a result, viruses were capable of more effective transmission and replication. Around the world, most SARS-CoV-2 isolates have the D614G mutation (Zhang et al., 2020).

Until now, many notable variants of SARS-CoV-2 have been reported, among which four have been declared as variants of concern (VOCs) by the WHO (Khan et al., 2021a). These VOCs are classified into four lineages: alpha (*a*), beta (*ß*), gamma (γ), and delta (∆) variants. Among these, alpha (B1.1.7) emerged in the United Kingdom in September 2020 and increased transmissibility and virulence was reported due to mutations in the spike protein (Khan et al., 2021b). Mutations N501Y and P681H were reported to be associated with higher infectivity. On the other hand, in South Africa, beta (lineage B.1.351) emerged, with notable mutations, mostly K417N, E484K, and N501Y, affecting the transmission speed and antigenicity. Similarly, gamma (lineage P.1) emerged in Brazil in the same year with similar mutations K417T, E484K, and N501Y as the B.1.351 variant. Delta (lineage B1.617.2) and B.1.617 were first discovered in India in late 2020 and then spread to other countries (Khan et al., 2020a; Khan et al., 2021c). These variations in the structural protein led to antigenicity drift and increased virus transmission and pathogenesis. Studies reported that these variants mainly increase the number of hydrogen bonds and salt bridges, consequently increasing the binding affinity toward the host receptor ACE2. These mutations also reduce the efficacy of the antibodies immune response *via* multiple deletions at the NTD (N-terminal domain) of the spike protein (Khan et al., 2020a; Khan et al., 2021b; Khan et al., 2021c). They are also reported to reduce efficacy (Khan et al., 2021a).

Mutational alterations in amino acids are anticipated to influence the structure and function of the related proteins. Therefore, it is imperative to discover the mutational landscape while creating novel antiviral therapies, and hence it is important to determine the mutations that have been observed in the spike protein polyprotein and subsequent influence in the protein structure and interaction with the host body. Therefore, the current work seeks to identify the mutations found in the spike protein to forecast the structural changes of SARS-COV-2 spike protein owing to the mutations and determine the signature pattern. Mutational analysis of the spike protein in SARS-CoV-2 isolates from Pakistan was performed using Global Initiative on Sharing All Influenza Data (GISAID), and mutations were identified. The impact of these substitutions on the structure and function of the spike protein was then investigated using various structural modeling tools. The current study provides the basis for therapeutics development to control the SARS-CoV-2 pandemic in Pakistan and worldwide.

## 2 Materials and methods

### 2.1 Mutations identification

The NCBI database was used for the retrieval of SARS-COV-2 Pakistan-specific sequences (https://www.ncbi.nlm.nih.gov/) (Pruitt et al., 2005), and then the “CoVsurver” module of the GISAID database (https://www.gisaid.org/epiflu-applications/covsurver-mutations-app/) was used for single nucleotide substitution in the spike protein (Shu and McCauley, 2017). The server query requires a sequence in the FASTA format. By comparing with reference sequence hCoV-19/Wuhan/WIV04/2019 (accession no MN996528.1), the server identified novel mutations along with substitute residue positions on the spike protein sequence (Okada et al., 2020).

### 2.2 Spike protein sequence retrieval and preparation

The spike protein sequence with a full length of (1273aa) was collected in the FASTA format from UniProt (https://www.uniprot.org/) (Consortium, 2019). The 3D structure of the spike glycoprotein was downloaded from RCSB using PDB ID: 6XRA (Cai et al., 2020). The missing residues were modeled using the comparative modeling method implemented in Modeler embedded in Chimera (Pettersen et al., 2004; Goddard et al., 2005; Webb and Sali, 2016; Webb and Sali, 2021). The final structure was refined and minimized prior to further analysis using Galaxy Refine (Ko et al., 2012; Heo et al., 2013).

### 2.3 Domain identification and mutation mapping

All the retrieved variants were classified based on the domain’s information, retrieved from UniProt’s Family and Domain option (https://www.uniprot.org/uniprot/P0DTC2#family_and_domains). Mutations identified through comparative analysis were mapped to their respective domains.

### 2.4 Impact of mutations on spike protein’s stability

#### 2.4.1 Structure-based analysis

The mCSM server (http://biosig.unimelb.edu.au/mcsm/stability) was used to accurately predict the impact of mutations on the protein stability and interaction by using graph-based signatures. For each mutation, relative solvent accessibility (RSA) and DDG values were computed (Pires et al., 2014). Furthermore, DynaMut2 (http://biosig.unimelb.edu.au/dynamut2/submit_prediction) was used for assessing the impact of the mutation on protein stability and dynamics by using the normal mode analysis (NMA) method. Predicted Gibbs free energy (ΔΔG) values of mutants less than zero (0) were classified as destabilizing, while those greater than 0 were classified as stabilizing (Rodrigues et al., 2021).

#### 2.4.2 Sequence-based analysis

Determination of the impact of mutations on protein stability and interactions is key in understanding any distortion in protein structure and its related function. Thus, it is essential to accurately predict protein stability changes upon mutation. The I-Mutant server (http://folding.biofold.org/i-mutant/i-mutant2.0.html) (Casadio et al., 2008) was used for predicting protein stability changes upon mutations in the spike protein of SARS-COV-2 based on sequence information, using, by default, pH and temperature. The server predicted the free energy value changes of mutations or DDG. The server query requires a wild-type residue position and mutated protein sequence for predicting the effect of amino acid substitution on the protein. Positive DDG values (+) indicate high stability, while negative values (−) indicate low stability.

### 2.5 Impact of mutation on protein binding

#### 2.5.1 Wild-type structure retrieval and mutant modeling

Structures of spike protein subunits of SARS-COV-2 were collected from RCSB with host receptor, the wild-type structure of RBD–ACE2 (PDB ID: 6M0J), and NTD–monoclonal antibody (4A8) (PDB ID: 7C2L) in a PDB file. The wild-type RBD and NTD structures were used as templates for mutant modeling using Chimera software. After each mutant modeling, each structure was prepared using an AMBER force field; hydrogen and charges were fixed. Finally, the prepared structures were minimized.

#### 2.5.2 Docking of RDB domain with human ACE2 and monoclonal antibody with NTD

To check the binding efficiency of wild-type and mutant RBD with the human ACE2 receptor, the PyDock (https://life.bsc.es/pid/pydockweb) algorithm was used for molecular docking (Cheng et al., 2007). PyDock is a rigid-body docking method of the protein–protein interaction that uses FTDock for sampling. The server provides the top 10 models of a complex with the best scoring. Scoring is based on an empirical potential composed of electrostatics and desolvation terms, with a limited contribution from van der Waals energy (Cheng et al., 2007). The same server was also used to dock wild-type and mutant NTD with monoclonal antibodies. The server provided the top 100 models of each complex. On the basis of the lowest energy scoring complex, each interaction was noted and top rank models were selected for each wild and mutant complex. The interaction result was explored through PDBsum (http://www.ebi.ac.uk/thornton-srv/databases/pdbsum/Generate.html).

### 2.6 Prediction of dissociation constant

Determination of the dissociation constant (K_D_) is an important parameter to estimate the strength of biological macromolecules association. Thus, herein, to determine K_D_, we used the PROtein binDIng enerGY prediction (PRODIGY) server (Xue et al., 2016).

### 2.7 Dynamics of the wild and mutant (RBD and NTD) complexes

To reveal the dynamic variations of the wild and mutant complexes, an all-atom simulation of 50 ns was achieved by using AMBER20 employing FF19SB (Salomon-Ferrer et al., 2013; Salomon‐Ferrer et al., 2013). Abbas et al. (2021) supplied complete details on system preparation and performing MD simulation production runs (Khan et al., 2021b). Briefly, a TIP3P water box was used for solvation, followed by the neutralization by adding Na^+^ ions. Gentle minimization using 6,000 and 3,000 steps of steepest descent and conjugate gradient algorithms was achieved, and heating at 300K, followed by the equilibration, was completed. Lastly, a production run at a time scale of 50 ns was executed. Simulation trajectories were analyzed using the CPPTRAJ and PTRAJ modules of AMBER (Roe and Cheatham, 2013).

### 2.8 Estimation of binding free energy

The AMBER MMGBSA. py module was used to calculate the binding free energies of the mutant and wild-type complexes (Hou et al., 2011). Energy elements such as electrostatic energy, van der Waals energy (vdW), and polar and non-polar solvation energies were calculated using the MM/GBSA technique, which is extensively used in many research studies. (Khan et al., 2018; Khan et al., 2020a; Khan et al., 2020b; Khan et al., 2020c; Khan et al., 2020d; Hussain et al., 2020; Khan et al., 2021d). Entropy was not calculated because it is computationally costly, time consuming, and prone to a higher number of inaccuracies. The energy terms were also mathematically processed to estimate the system’s net binding free energy, using the following equation:

$${\prime \prime ∆G}_{net\;binding\;energy}={∆G}_{complex\;binding\;energy}-{\left[\;{∆G}_{receptor\;binding\;energy}+{∆G}_{ligand\;binding\;energy}\right]}^{\prime \prime }.$$

Each of the aforementioned components of net binding energy can be split as follows:

$$\prime \prime G=G_{bonded}+G_{van\;der\;waals}+G_{polar\;solvation\;energy}+G_{non-polar\;solvation\;energy}\prime \prime .$$

## 3 Results

### 3.1 Mutation identification

**FIGURE 1:**
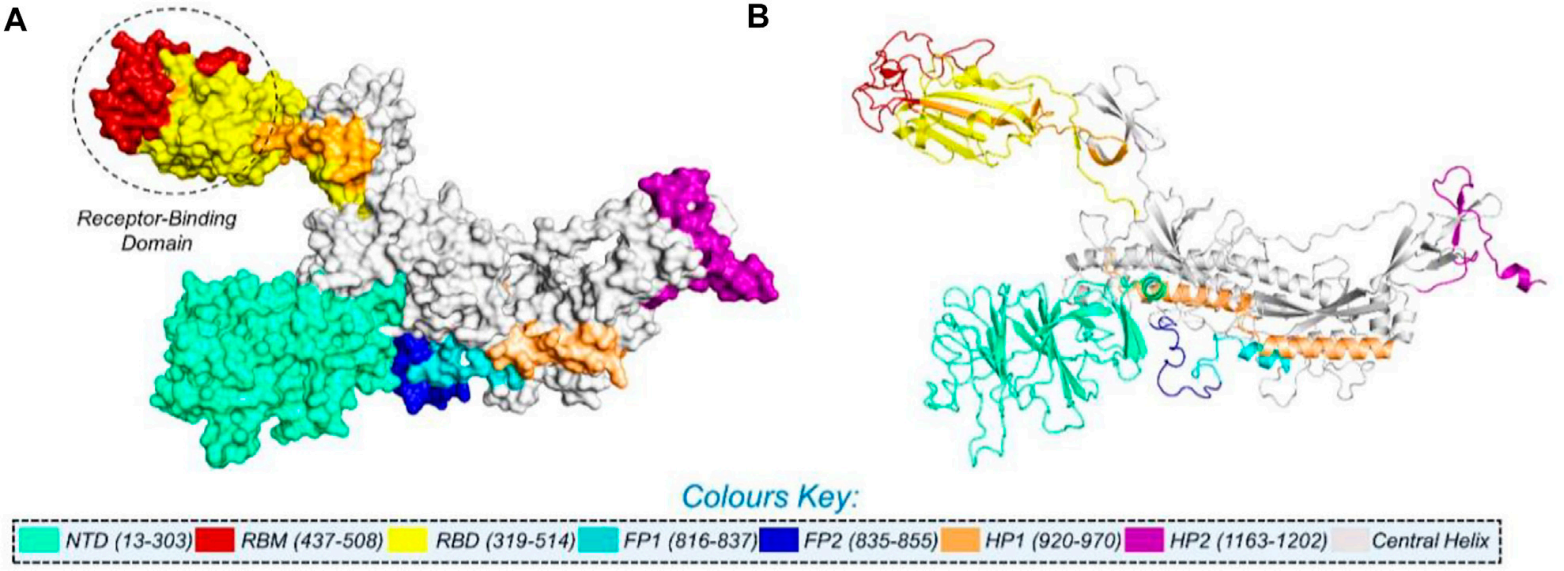
Multi-domain organization of the full-length spike protein from SARS-CoV-2. **(A)** Surface representation of the spike protein and **(B)** presents the cartoon view. The RBD is encircled in panel **(A)**.

NCBI GISAID databases were used to collect and analyze 215 genomes of Pakistani isolates of novel coronavirus 2019. The spike protein sequence in the FASTA format of each isolate was then analyzed by CoVsurver of the GISAID database (https://www.gisaid.org/epiflu-applications/covsurver-mutations-app/) to identify single amino acid changes throughout the whole protein sequence (1273aa). There were 21 different types of single amino acid substitutions (single mutant) identified in the protein. The mutations include H49Y, L54F, N74K, D80Y, R102S, G142A, G181V, A222V, G261R, G446V, D614G, D614A, V622F, Q67H, S813N, C840V, A846V, A890T, S943T, A1078S, and T117I. Of these, five mutations stabilized the structure and 16 mutations destabilized it (Table 1). According to these, the identified H49Y, L54F, N74K, D80Y, R102S, G142A, G181V, A222V, and G261R mutants fall into 13-303 BetaCoV S1-NTD, whereas S813N, A890T, A1078S, and T1117I were reported in the CoV_S2 domain, which starts from position 711aa and is extended to the 1232aa position. Two variants, C840V and A846V, were reported specifically in the fusion peptide 2 at 835–855aa. One variant, G446V, was found to be precisely in the 319-541 receptor-binding domain, and another one, variant S943T, in the 920-970 heptad repeat 1. The rest of the variants D614G, D614A, V622F, and Q677H are observed to be in the central helix (Isabel et al., 2020). The general structure and domain organization of spike protein are given in Figure 1.

**TABLE 1:** Mutation classification on the basis of domains and sub-peptides.

Index	Mutation	RSA (%)	Predicted ΔΔG (mCSM)	Outcome
1	D614G	95.7	−.665	Destabilizing
2	G446V	88.5	−.277	Destabilizing
3	T1117I	67.5	.082	Stabilizing
4	H49Y	38.2	.945	Stabilizing
5	G181V	100.0	−.488	Destabilizing
6	A890T	89.9	−.309	Destabilizing
7	G261R	41.1	−.309	Destabilizing
8	L54F	23.5	−1.298	Destabilizing
9	S813N	40.2	−.46	Destabilizing
10	D614A	95.7	−.445	Destabilizing
11	D80Y	70.2	.091	Stabilizing
12	G142A	0.0	−.908	Destabilizing
13	A1078S	2.8	−1.33	Destabilizing
14	A846V	26.9	−.127	Destabilizing
15	S943T	101.3	−.306	Destabilizing
16	V622F	35.5	−.821	Destabilizing
17	R102S	9.0	−1.345	Destabilizing
18	A222V	2.2	.096	Stabilizing
19	Q677H	95.4	−.179	Destabilizing
20	N74K	91.9	.096	Stabilizing
21	C840V	39.9	−.418	Destabilizing

#### 3.2.1 Structure-based analysis of mutations using mCSM

In this study, we analyzed 21 mutations in the spike protein of SAR-CoV-2 using “mCSM.” The impact of each mutation identified by the GISAID is given in Table 2.

**TABLE 2:** Analysis of spike protein stability changes upon mutations by mCSM.

Domain	Mutations	Protein
S1-NTD domain	H49Y, L54F, N74K, D80Y, R102S, G142A, G181V, A222V, G261R	Spike protein
S1 CTD of RBD	G446V	Spike protein
Heptad repeat 1	S943T	Spike protein
Central helices	D614G, D614A, V622F, Q677H	Spike protein
S2 domain	S813N, A890T, A1078S, T1117I, C840V, A846V	Spike protein
Fusion peptide	C840V, A846V	Spike protein

##### 3.2.1.1 S1 domain

**FIGURE 2:**
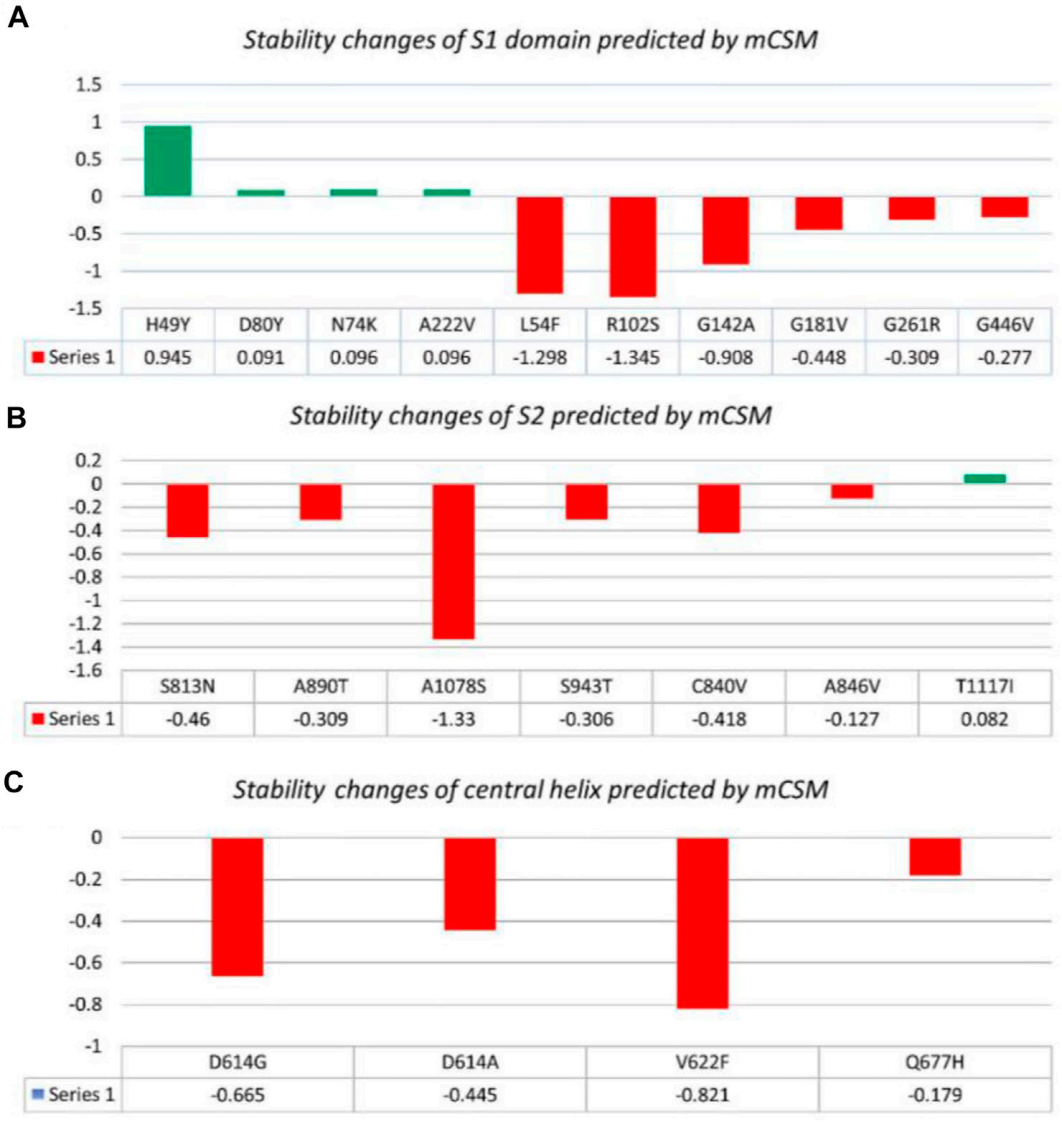
Analysis of spike protein stability changes upon mutation by mCSM. **(A)** Mutation effect prediction in the S1 domain using mCSM. **(B)** Mutation effect prediction in the S2 domain using mCSM. **(C)** Mutation effect prediction in the central helix using mCSM. The green color bar represents stabilizing, while the red color bars represent destabilizing mutations.

Nine mutations were found in S1 (NTD), including, H49Y, L54F, N74K, D80Y, G142A, G181V, A222V, and G261R. Among four of them, mainly H49Y “predicted ΔΔG value” (.945), D80Y (.091), N74K (.096), and A222V (.096) were responsible for stabilizing the S protein structure and the rest of the mutants. L54F (−1.298), R102S (−1.345), G142A (−.908), G181V (−.488), and G261R (−.309) were found to be destabilizing. For instance, the H49Y mutation was also previously reported to stabilize the structure and is responsible for enhanced transmission as also reported in the B.1.618 variant (Sixto-López et al., 2021). Similarly, the L54F mutation in association with D614G was reported to synergistically increase the stability but not alone (Laha et al., 2020). The N74K mutant occurred at the glycosylation site and was reported to affect the glycosylation (Li et al., 2020). The D80Y mutation was reported in the 20A.EU1 variant, and it was also reported that this strain originated in Spain and traveled to other parts of Europe (Hodcroft et al., 2021a). G142A is a novel mutation and only reported in new Pakistani samples. On the other hand, the G181V substitution, also previously reported in the P.1 variant, is located in the NTD of the spike protein and does not have a crucial role in antigenic modification since amino acid substitutions at position 181 have never conferred resistance to neutralizing human monoclonal antibodies. In *in vitro* testing, it gives the same antibody titer (Imai et al., 2021). The A222V reported in the GV clade in Spain was associated with rapid transmission, but no association of evading the antibodies or vaccine was reported (Bartolini et al., 2020). G261R that was reported to increase the stability of spike protein is also reported in B.1.36 and B.1.36.16 variants of Bangladesh (Saha et al., 2021). The S1 RBD contains G446V (−.277); destabilizing the structure of the respective protein was also previously reported to confer resistance to four different types of sera tested (Figure 2A) (Liu et al., 2021).

##### 3.2.1.2 S2 domain

The S2 domain contains S813N, A890T, A1078S, and T1117I; some subunits/peptides such as heptad repeat 1 contain S943T and fusion peptide 2 contain C840V and A846V, in which S813N (−.46), A890T (−.309), A1078S (−1.33), S943T (−.306), C840V (−.418), and A846V (−.127) were analyzed to be destabilizing, while T1117I (.082) stabilizes the S protein structure (Figure 2B). The S813N mutation is reported in B.1.1.7 and P.1 variants, but no functional consequence was reported (Di Giallonardo et al., 2021). A890T is a newly reported mutation and is not reported by any literature. A1078S is reported to increase the infectivity by 1.2%, while it significantly affects the sequence of the antigenic epitope, thus hindering the design of antigenic vaccines (Xu et al., 2020; Tegally et al., 2021). A study reported the T1117I mutation to impact the viral oligomerization but showed no association with vaccines or antibody evasion (Molina-Mora et al., 2021). The S2 domain mainly facilitates viral entry into the host cell, where the virus initiates infection.

##### 3.2.1.3 Central helices

Central helices contain D614G (−.665), D614A (−.445), V622F (−.821), and Q677H (−.179), which were analyzed to destabilize the S protein (Figure 2C). The D614G/A mutation was reported to increase viral fitness and infectivity (Plante et al., 2021). V622F is a novel variant only reported in Pakistani samples. The Q677H mutation also reported in B.1.429 is associated with increased cases (Hodcroft et al., 2021b).

#### 3.2.2 Structure-based analysis of mutations using DynaMut2

Additionally, the DynaMut2 server was also used for mutation verification and their related effects on the spike protein structure and dynamics. Out of 21 mutations, seven were found to be responsible for stabilizing, and the remaining mutants (14) were found to destabilize the protein (Table 3).

**TABLE 3:** Analysis of spike protein stability changes upon mutations by DynaMut2.

Index	Mutation	Predicted ΔΔG (DynaMut2)	Outcome
1	D614G	−.22 kcal/mol	Destabilizing
2	G446V	−.75 kcal/mol	Destabilizing
3	T1117I	.43 kcal/mol	Stabilizing
4	H49Y	1.27 kcal/mol	Stabilizing
5	G181V	−1.07 kcal/mol	Destabilizing
6	A890T	.16 kcal/mol	Stabilizing
7	G261R	−.51 kcal/mol	Destabilizing
8	L54F	−.69 kcal/mol	Destabilizing
9	S813N	−.48 kcal/mol	Destabilizing
10	D614A	−.4 kcal/mol	Destabilizing
11	D80Y	.16 kcal/mol	Stabilizing
12	G142A	−.28 kcal/mol	Destabilizing
13	A1078S	−.7 kcal/mol	Destabilizing
14	A846V	−.73 kcal/mol	Destabilizing
15	S943T	−.49 kcal/mol	Destabilizing
16	V622F	−.3 kcal/mol	Destabilizing
17	R102S	−1.02 kcal/mol	Destabilizing
18	A222V	.17 kcal/mol	Stabilizing
19	Q677H	.02 kcal/mol	Stabilizing
20	N74K	.03 kcal/mol	Stabilizing
21	C840V	−.59 kcal/mol	Destabilizing

##### 3.2.2.1 S1 domain

**FIGURE 3:**
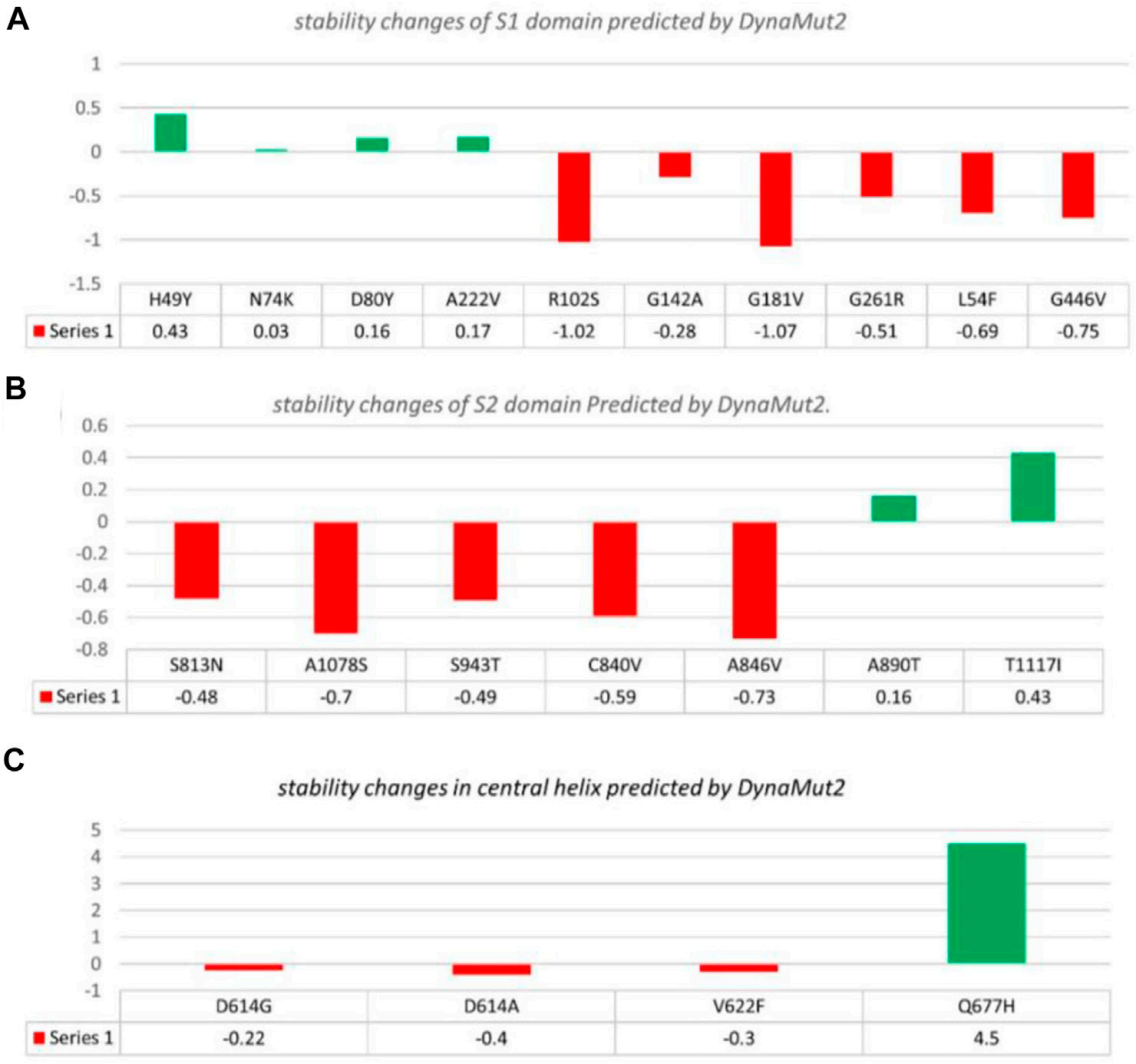
Analysis of spike protein stability changes upon mutations by DynaMut 2.0. **(A)** Mutation effect prediction in the S1 domain using DynaMut 2.0. **(B)** Mutation effect prediction in the S2 domain using DynaMut, while **(C)** show the effect of mutation in the central helix predicted by DynaMut 2.0.

In the S1 domain, NTD H49Y (.43), N74K (.03), D80Y (.16), and A222V (.17) were analyzed to be stabilizing, while NTD R102S (−1.02), G142A (−.28), G181V (−1.07), G261R (−.51), L54F (−.69), and RBD G446V (−.75) were found to destabilize the S protein (Figure 3A).

##### 3.2.2.2 S2 domain

The S2 domain contains S813N (−.48), A1078S (−.7), S943T (−.49), C840V (−.59), and A846V (−.73), which were found to be destabilizing, while A890T (.16) and T1117I (.43) were found to stabilize the S protein (Figure 3B).

##### 3.2.2.3 Central helices

The central helices contain D614G (−.22), D614A (−.4), and V622F (−.3), which were analyzed as destabilizing, while Q677H (.02) was found to stabilize the S protein (Figure 3C).

#### 3.2.3 Sequenced-based analysis of mutations

**FIGURE 4:**
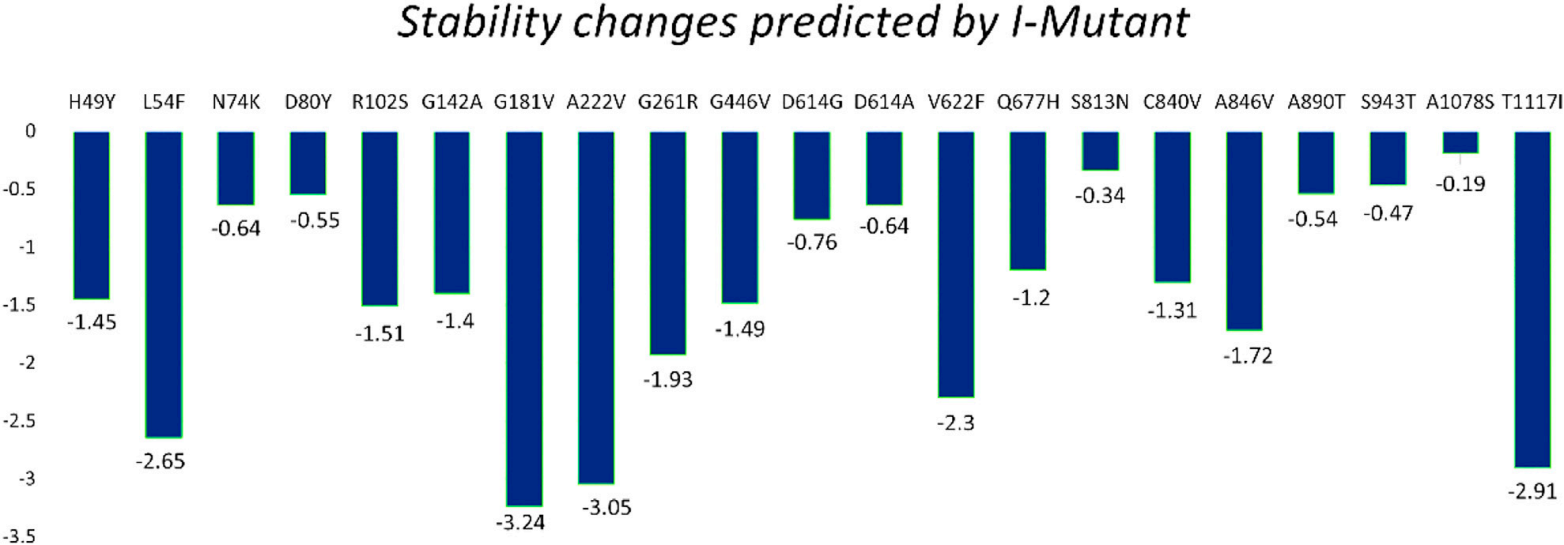
Spike protein mutation effect on structural stability as predicted by I-Mutant.

The I-Mutant server shows that these all identified mutations (21) in the spike glycoprotein decreased structural stability (Table 4). In this, the S1 domain contains H49Y (−1.45), L54F (−2.65), N74K (−.64), D80Y (−.55), R102S (−1.51), G142A (−1.40), G181V (−3.24), A222V (−3.05), G261R (−1.93), and G446V (−1.49). The central helices contain D614G (−.76), D614A (−.64), V622F (−2.30), and Q677H (−1.20). The mutations S813N (−.34), C840V (−1.31), A846V (−1.72), A890T (−.54), S943T (−.47), A1078S (−.19), and T1117I (−2.91) were found in the S2 domain (Figure 4).

**TABLE 4:** Analysis of spike protein stability changes upon mutations by I-Mutant2.0.

Index	Mutation	Predicted DDG I-Mutant2.0	Outcome
1	H49Y	−1.45	Decrease stability
2	L54F	−2.65	Decrease stability
3	N74K	−.64	Decrease stability
4	D80Y	−.55	Decrease stability
5	R102S	−1.51	Decrease stability
6	G142A	−1.40	Decrease stability
7	G181V	−3.24	Decrease stability
8	A222V	−3.05	Decrease stability
9	G261R	−1.93	Decrease stability
10	G446V	−1.49	Decrease stability
11	D614G	−.76	Decrease stability
12	D614A	−.64	Decrease stability
13	V622F	−2.30	Decrease stability
14	Q677H	−1.20	Decrease stability
15	S813N	−.34	Decrease stability
16	C840V	−1.31	Decrease stability
17	A846V	−1.72	Decrease stability
18	A890T	−.54	Decrease stability
19	S943T	−.47	Decrease stability
20	A1078S	−.19	Decrease stability
21	T1117I	−2.91	Decrease stability

### 3.3 Molecular docking of RBD and NTD with ACE2 and mAb

The 3D structures of spike protein RBD–ACE2 and NTD–mAbs were collected from RCSB using PDB ID: 6M0J and PDB ID: 7C2L. Furthermore, these experimentally reported structures of S1 regions (NTD and RBD) were used for mutant modeling using Chimera software. The NTD domain mutants include H49Y, L54F, N74K, D80Y, R102S, G142A, G181V, A222V, and G261R, while G446V was modeled in the wild-type RBD domain.

#### 3.3.1 Wild and mutant RBD docking with ACE2

**FIGURE 5:**
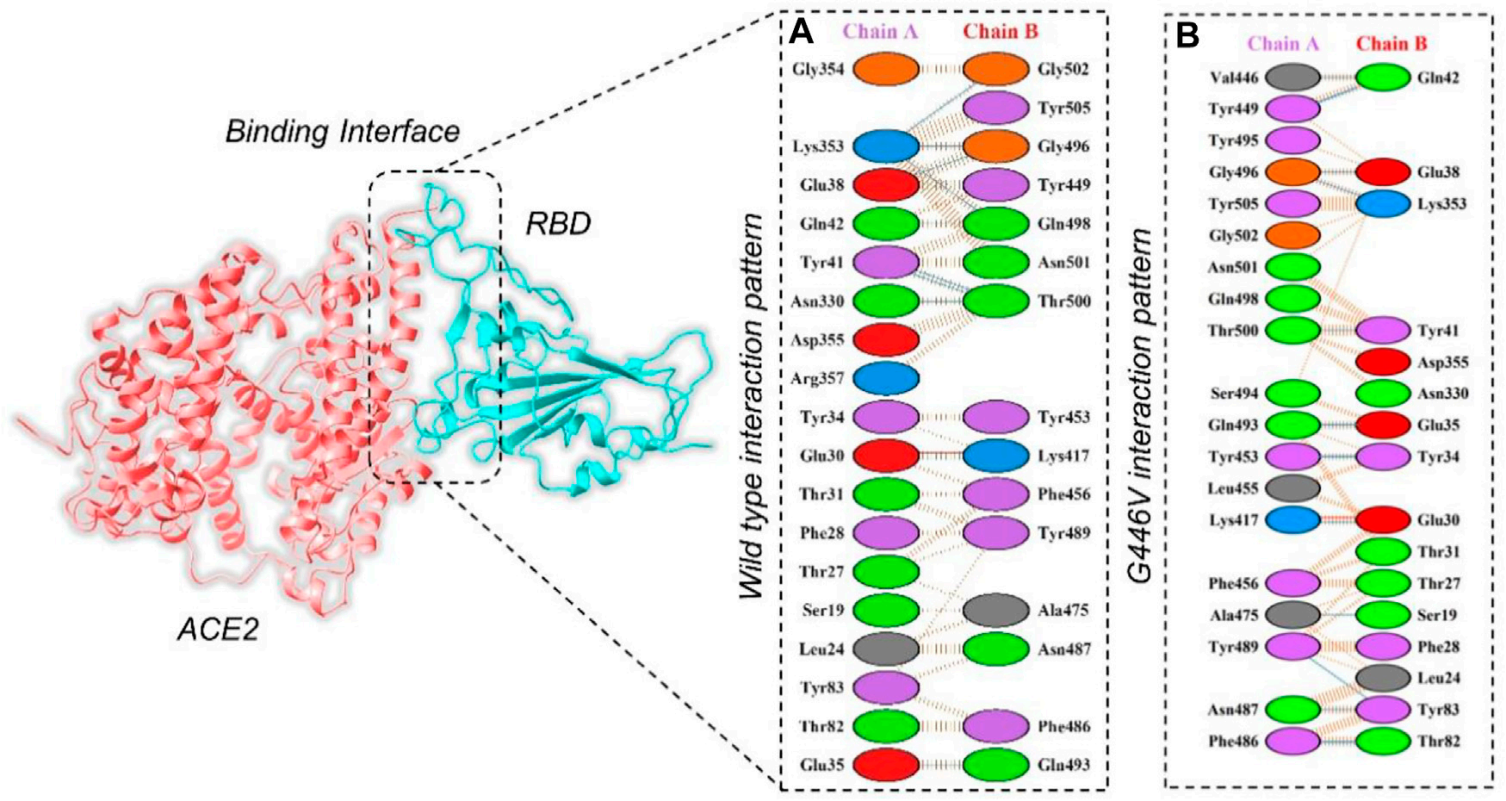
Representation of mutant G446V-RBD binding interaction with the ACE2 receptor. **(A)** Binding of wild-type RBD–ACE2 and **(B)** binding interface of G446V–ACE2 along with its stick representation of the key hydrogen interactions.

By using the PyDock server (https://life.bsc.es/pid/pydockweb), we docked the spike (S1) RBD with the human ACE2 receptor to check the effect of 10 identified single amino acid substitutions in the S1 subunit of SARS-COV-2 of Pakistani isolates on the binding efficiency of RBD and NTD domains with human ACE2. The mutants were generated using Chimera software. By using the PyDock server, ACE2 was docked with a wild-type spike (RBD); and then, PDBsum was used (http://www.ebi.ac.uk/thornton-srv/databases/pdbsum/Generate.html) to explore interaction analysis of complex. The PDBsum interaction analysis revealed that two salt bridges, 10 hydrogen bonds, and 145 non-bonded contacts were formed. The complex formed 10 double hydrogen bonds between Asn487–Gln24, Lys417–Asp30, Gly446–Gln42, Gln493–Glu35, Tyr449–Asp38, Tyr449–Gln42, Asn487–Tyr83, Gly502–Lys353, Gly496–Lys353, Tyr505–Arg393, and Thr500–Tyr41 residues. The two salt bridges formed between Lys417–Asp30 residues **(** Figure 5A **)**. The interaction analysis of the PyDock server revealed that the mutant (G446V) RBD–ACE2 complex forms 12 hydrogen bonds and 135 non-bonded contacts. The hydrogen bonds include Lys417–Glu30, Val446–Gln42, Ala475–Ser19, Asn487–Tyr83, Tyr489–Tyr83, Gly496–Glu38, Gly496–Lys353, Thr500–Tyr41, Ser494–Asp157, Tyr505–Tyr158, Lys417–Asn250, and Glu406–Ser254 residues, while a salt bridge was formed between Lys417 and Glu30 **(** Figure 5B **)**. Similar findings were also reported previously by different studies; thus, our results are consistent and accurate (Khan et al., 2020a; Khan et al., 2021b; Khan et al., 2021c).

#### 3.3.2 Wild-type and mutant NTD docking with monoclonal antibody

**FIGURE 6:**
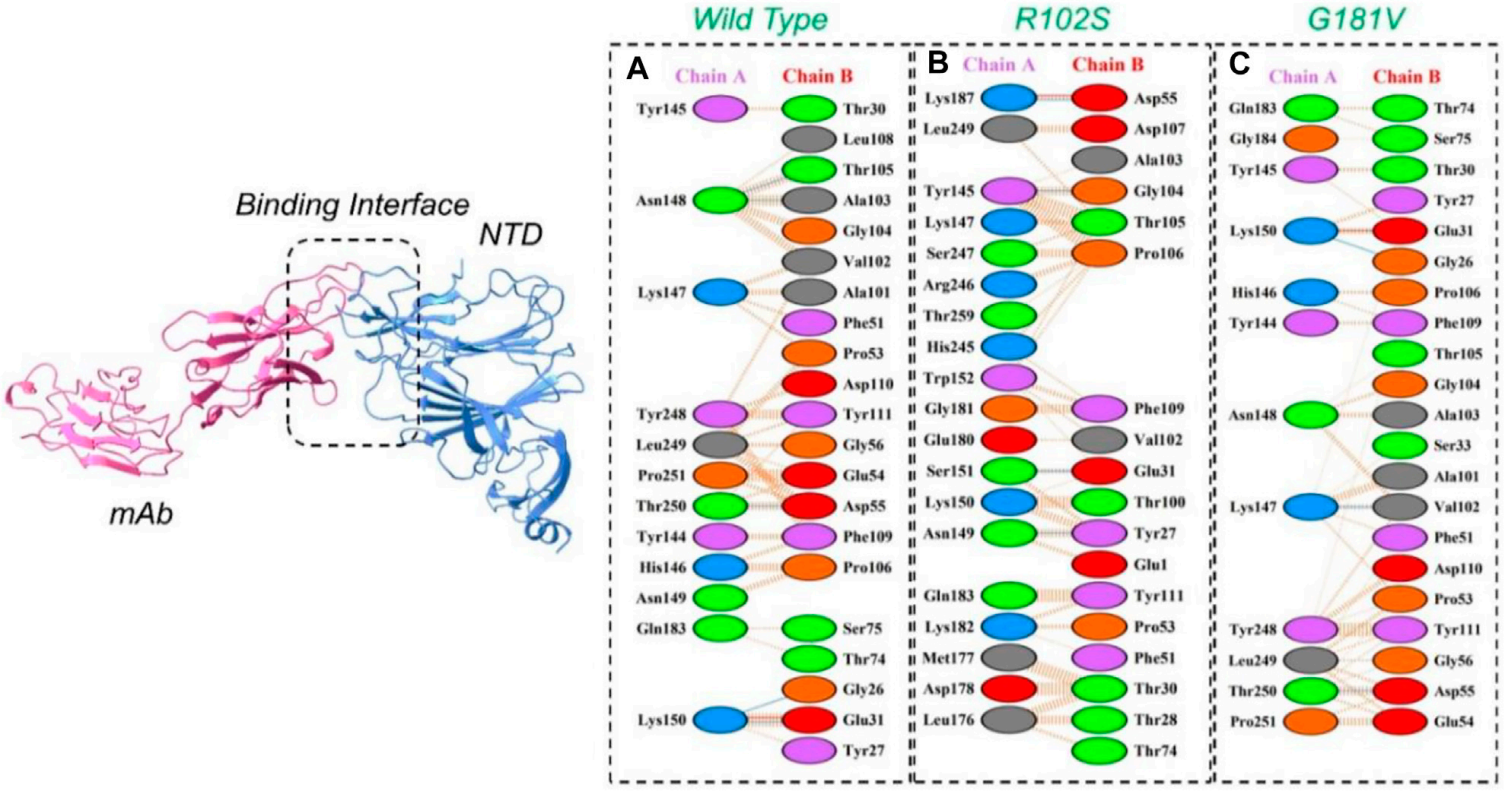
Representation of wild type, R102S, and G181V NTD of spike binding to neutralizing monoclonal antibody. **(A)** Binding of wild-type NTD–mAbs, **(B)** binding of R102S NTD–mAbs, **(C)** binding of G181V-NTD–mAbs. The binding interface along with 2D interactions’ representation of the key hydrogen and salt bridges are shown.

The PyDock server was used to dock wild-type NTD with mAbs. The PyDock docking score for the wild-type NTD–mAbs complex was as follows: electrostatic score (−36.092), desolvation (−14.139), and VdW (95.823). The interaction analysis revealed that the interaction forms one salt bridge, four hydrogen bonds, and 265 non-bonded contacts. The hydrogen bonds formed by the wild NTD–mAbs complex include Asn149–Tyr27, Ser151–Glu31, and Lys187–Asp55 and Tyr145–Gly104 residues. The single salt bridge forms between Lys187 and Asp55 in the wild NTD–mAbs complex **(** Figure 6A **)**. On the other hand, with the docking score −112–91 kcal/mol, the R102S–mAbs showed that the complex formed one salt bridge, four hydrogen bonds, and 268 non-bonded contacts **(** Figure 6B **)**. Moreover, the PyDock docking score for the mutant (G181V) NTD–ACE2 complex was as follows: electrostatics (−32.118), desolvation (8.856), and VdW (68.920). The interaction analysis of mutant G181V–mAbs revealed that both structures formed one salt bridge, three hydrogen bonds, and 305 non-bonded contacts. The hydrogen bonds formed between Lys150–Gly26, Thr250–Asp55, and Lys147–Val102 residues. The salt bridge forms between Lys150 and Glu31 residues **(** Figure 6C **)**. The interaction analysis of H49Y NTD–mAbs by PDBsum revealed that the interaction formed one salt bridge, four hydrogen bonds, and 266 non-bonded contacts. The hydrogen bonds formed between Asn149–Tyr27, Ser151–Glu31, and Lys187–Asp55 and Tyr145–Gly104 residues. The single salt bridge forms between Lys187 and Asp55 residues. The PyDock interaction analysis of mutant (L54F) with mAbs by PDBsum analysis revealed that both structures form one salt bridge, four hydrogen bonds, and 268 non-bonded contacts. The interaction analysis of this complex exhibits a binding pattern similar to the wild-type complex. The PDBsum analysis of mutant D80Y–mAbs revealed that both structures form one salt bridge, four hydrogen bonds, and 266 non-bonded contacts. In this complex the salt-bridges and hydrogen bonds determined the same pattern as the wild type complex, and the hydrogen binding pattern is similar to the wild complex.

The interaction analysis of mutant G142A–mAbs revealed that both structures form the same number of hydrogen bonds and salt bridges with hydrogen binding patterns similar to the wild-type NTD–mAbs complex. The interaction analysis of the mutant A222V–ACE2 receptor revealed that both structures form one salt bridge, four hydrogen bonds, and 264 non-bonded contacts. In this, the complex exhibits a binding pattern similar to that of the wild-type NTD–mAbs complex. The interaction analysis of mutant G261R–mAbs receptor revealed that the complex formed one salt bridge, four hydrogen bonds, and 268 non-bonded contacts. This complex shows a hydrogen binding pattern similar to that of the wild-type NTD–mAbs complex. In the PyDock docking score for mutant (N74K) NTD–mAbs, the complex shows electrostatics energy (−50.511), desolvation (−6.857), and vdW (121.522). The PDBsum analysis of mutant N74K–mAbs revealed that both structures formed three salt bridges, seven hydrogen bonds, and 274 non-bonded contacts. Among single hydrogen bonds, Ser71–Tyr27, Lys74–Tyr27, His69–Glu31, Asn185–Asp55, Tyr145–Thr74, Tyr145–Ser75, and His146–Ser75 residues were involved. The key residues Lys74–Glu1, His69–Glu31, and Lys147–Asp77 formed salt bridges. Consequently, the other mutations help the virus escape the neutralizing antibodies, while R102S, G181V, and G446V directly affect the binding. Thus, these three mutations, along with the wild type, were subjected to further analysis using a molecular dynamics simulation tool. The docking scores, including vdW, electrostatic, desolvation, and the docking scores of the wild type and mutant (RBDs and NTDs), are given in Table 5.

**TABLE 5:** Docking analysis of wild and mutants S1 Domain with ACE2 and mAb using PyDock.

Complex	Domain	Van der Waal energy	Electrostatic energy (Kcal/mol)	Desolvation energy	Total energy of complex
Wild–ACE2	RBD	−34.283	39.880	6,677	−132.785
G446V–ACE2	RBD	.227	−52.376	7.229	−145.782
Wild NTD–4A8	NTD	95.823	−36.092	−14.139	−115.008
G181V–4A8	NTD	68.920	−32.118	8.856	−108.441
H49Y–4A8	NTD	96.501	−37.088	−14.147	−117.195
L54F–4A8	NTD	95.927	−36.222	−14.119	−115.107
N74K–4A8	NTD	121.522	−50.511	−6.857	−119.575
D80Y–4A8	NTD	96.501	−37.088	−14.147	−115.944
R102S–4A8	NTD	95.301	−33.932	−14.149	−112.910
G142A–4A8	NTD	96.656	−36.262	−14.121	−115.076
A222V–4A8	NTD	94.499	−35.995	−14.152	−115.056
G261R–4A8	NTD	97.487	−40.846	−16.486	−121.942

### 3.4 Dynamic features of the wild-type and mutant complexes

**FIGURE 7:**
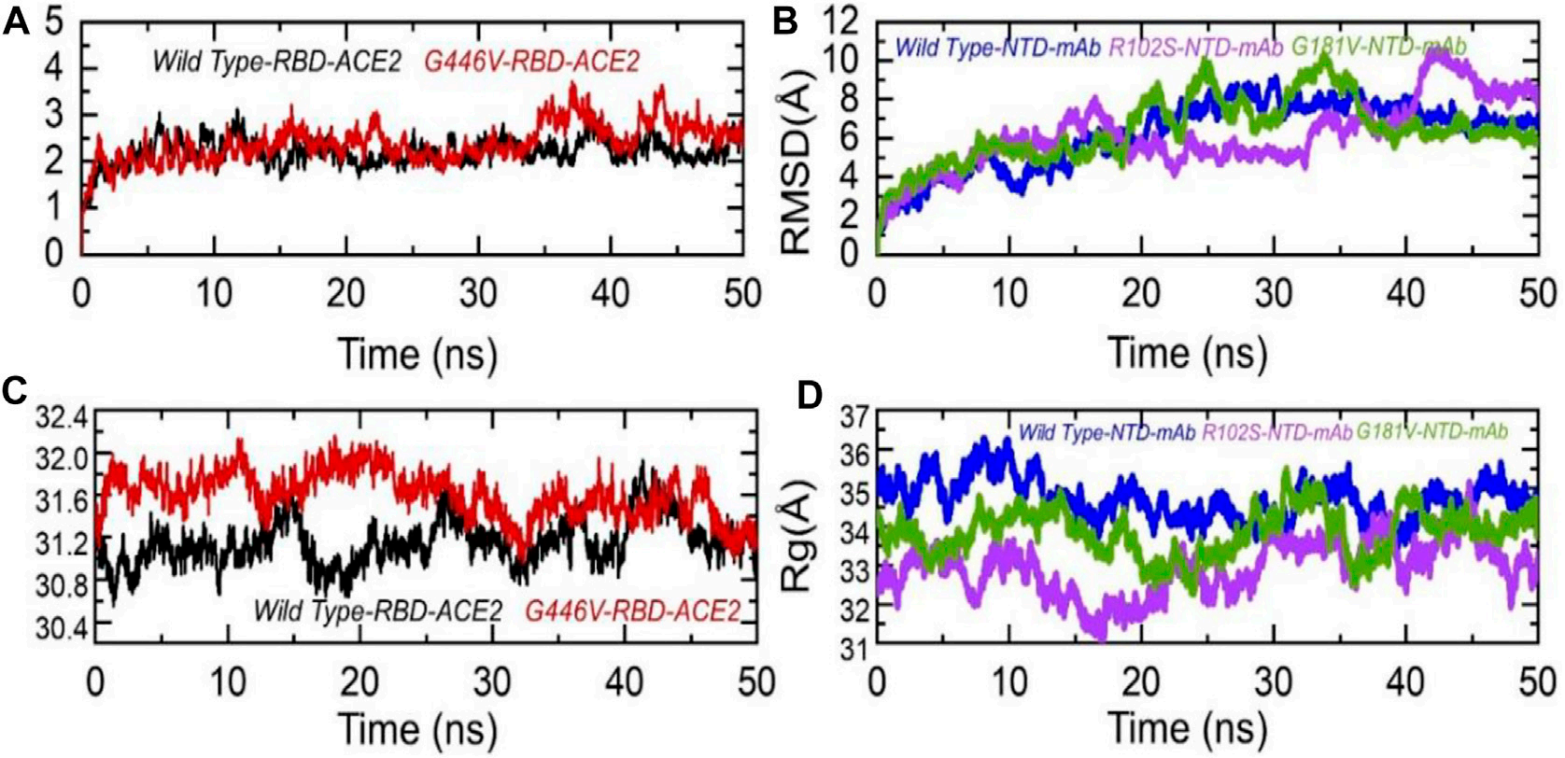
Dynamic stability and compactness of the wild-type and mutant complexes (RBD and NTD). **(A)** RMSDs of wild-type RBD–ACE2 and G446V-RBD–ACE2; **(B)** RMSDs of wild-type NTD–mAb, R102S-NTD–mAb, and G181V-NTD–mAb; **(C)** Rg(s) of wild-type RBD–ACE2 and G446V-RBD–ACE2; and**(D)** Rg(s) of wild-type NTD–mAb, R102S-NTD–mAb, and G181V-NTD–mAb complexes.

**FIGURE 8:**
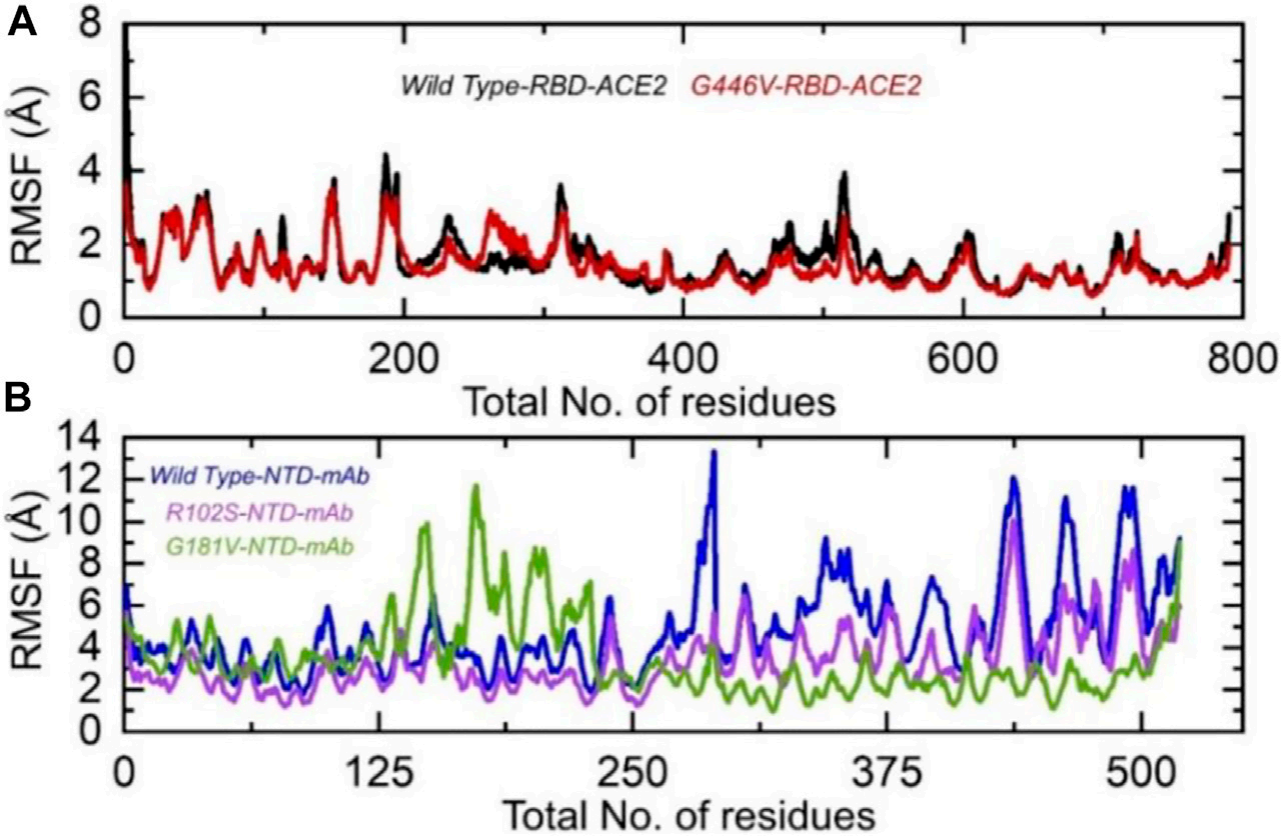
Residual flexibility of the wild-type and mutant complexes (RBD and NTD). **(A)** RMSFs of wild-type RBD–ACE2 and G446V-RBD–ACE2 and **(B)** RMSFs of wild-type NTD–mAb, R102S-NTD–mAb, and G181V-NTD–mAb complexes.

To further see the structural stability and compactness of the deleterious mutations, we performed MD simulation. We calculated the stability as root mean square deviation (RMSD) and compactness as the radius of gyration (Rg) as a function of time. Comparison of the wild-type and G446V-RBD complexes revealed more comparable RMSD. During the first 30 ns, both the complexes remained more stable; however, the G446V-RBD complex converged during the last 20 ns. The average RMSD for both the complexes was 2.0 and 2.1 Å, respectively. For instance, the unstable behavior of the G446V justifies the radical function of the variants, as reported previously. The RMSD graphs of the wild-type RBD–ACE2 and G446V-RBD–ACE2 complexes are given in Figure 8A. On the other hand, we also calculated RMSD for the wild type, R102S and G181V-NTD in complex with monoclonal antibody (4A8). All the structures initially reported no convergence until 20 ns, until 50 ns at different time intervals; the RMSD increased and decreased. Overall, the wild type remained more stable despite a gradual increase in the RMSD value. The average RMSD for each complex was reported to be 6.7 Å, 6.52 Å, and 6.6 Å. The RMSDs of wild-type NTD–mAb, R102S-NTD–mAb, and G181V-NTD–mAb are given in Figure 7B.

Moreover, to see the structural compactness differences, a dynamic environment Rg for each complex was computed. Variations in the wild-type RBD–ACE2 and G446V-RBD–ACE2 complexes were observed during the first stage of simulation particularly between 0 and 30 ns. In this time period, the wild type remained more compact than the G446V complex. Then, after 30 ns, the G446V complex also achieved the compact state and remained comparable with the wild type. The average Rg(s) for both the complexes was reported to be 31.3 Å and 31.6 Å, respectively. Increase or decrease in the compactness is due to the binding and unbinding events that happened during the simulation, respectively. The Rg(s) of all the complexes are given in Figures 7C, D.

### 3.5 Residual flexibility estimation

Understanding the residual flexibility helps in assessing the biological mechanism of different molecules and plays an important role in enzyme engineering, drug designing, interface study, and peptide inhibitor discovery. Thus, herein, we also calculated residual flexibility as root mean square fluctuation (RMSF) using the simulation trajectories. The wild-type RBD–ACE2 and G446V-RBD–ACE2 demonstrated a similar pattern of flexibility, except for minor variations in different regions. As can be seen, the regions 190–200 and 450–500 displayed higher fluctuation in the wild type. This shows that the G446V mutations have induced stable binding and thus stabilized the fluctuations. The RMSF of the wild-type RBD–ACE2 and G446V-RBD–ACE2 complexes is given in Figure 8A. The calculation of the RMSF for the wild-type and mutant NTD in complex with mAb demonstrated a significant variation in the residual flexibility. The wild type provides an opportunity for better conformational optimization, thus binding the mAb more robustly than the variants (R102S and G181V) (Figure 8B). In conclusion, this shows that with a higher binding affinity toward host ACE2, the circulating strains in Pakistan also escape the monoclonal antibodies.

### 3.6 Hydrogen bonding analysis

**FIGURE 9:**
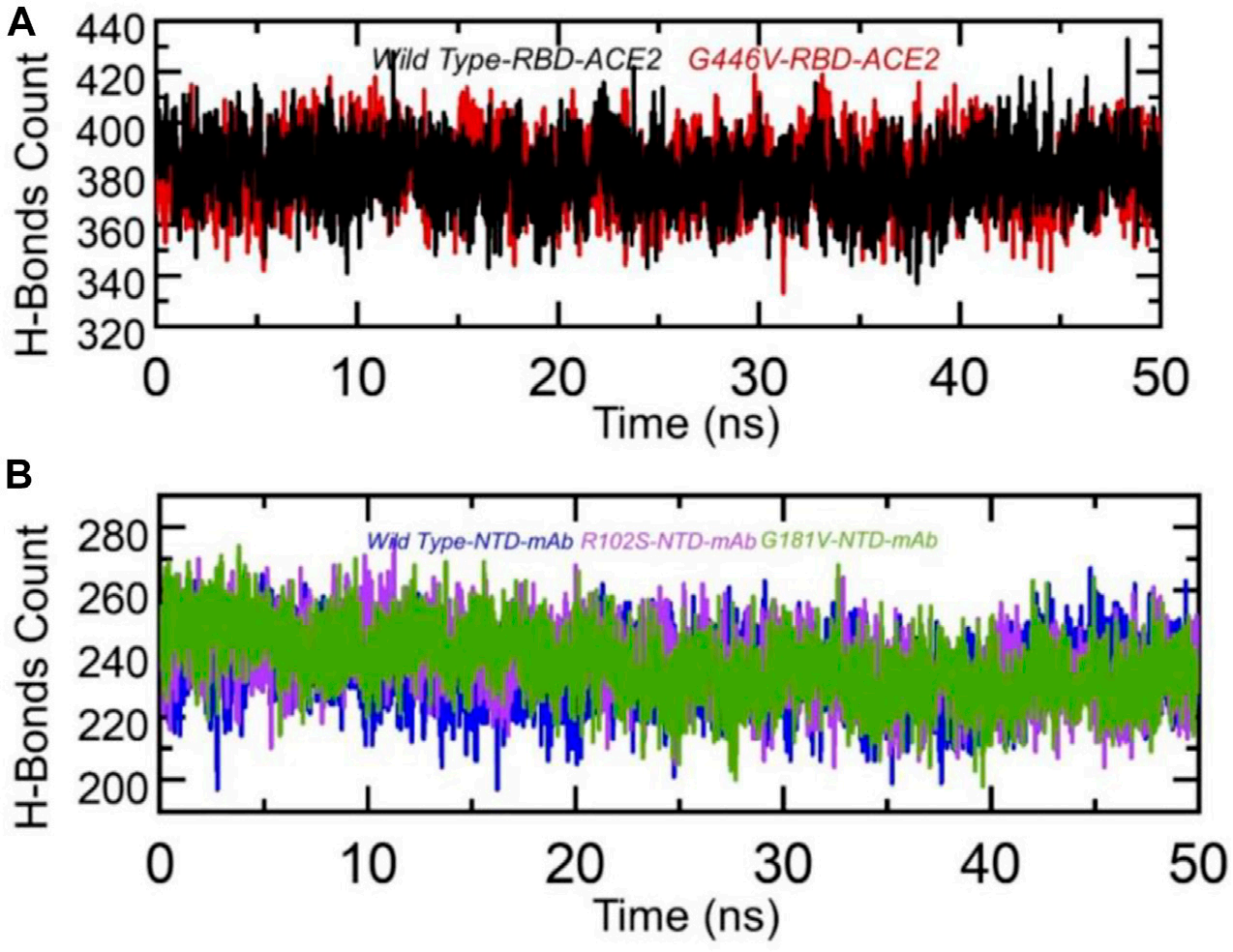
Hydrogen bonding analysis of the wild-type and mutant complexes (RBD and NTD). **(A)** H-bonds in wild-type RBD–ACE2 and G446V-RBD–ACE2 complexes and**(B)** H-bonds in wild-type NTD–mAb, R102S-NTD–mAb, and G181V-NTD–mAb complexes.

Hydrogen bonding is important to maintain the binding complex and has been previously reported by various studies to understanding the binding pattern of the wild type and other variants (Khan et al., 2020a; Khan et al., 2020d; Khan et al., 2021b; Khan et al., 2021c). The hydrogen bonds were calculated in complex as a function of time, and the average number of hydrogen bonds was estimated. In the wild-type RBD–ACE2, the average number of hydrogen bonds was 384, while in the G446V-RBD–ACE2 complex, the average number of hydrogen bonds was reported to be 388. This shows that the strain circulating in Pakistan and other regions with the G446V mutation induces higher binding *via* increment in the number of hydrogen bonds. To check the bonding differences in the wild-type NTD–mAb, R102S-NTD–mAb, and G181V-NTD–mAb complexes, we also calculated hydrogen bonds in each complex. In each complex, the number of hydrogen bonds was recorded as wild-type NTD–mAb (256), R102S-NTD–mAb (249), and G181V-NTD–mAb (247). Consequently, this shows that SARS-CoV-2 strains with these mutations reduce the binding of monoclonal antibodies and thus escape the immune response to increase the infectivity. The hydrogen bonds calculated in each complex are given in Figures 9A, B.

### 3.7 Binding free energy calculations

Estimation of the binding free energy defines the correction conformation and rescoring of the binding molecules. It is a widely used method to understand the binding of two or more molecules. To redefine the binding affinity predicted by docking, herein, we used the MM/GBSA approach using 2,500 frames. The binding free energy calculations revealed that G446V-RBD binds to the ACE2 receptor more strongly than the wild-type RBD. The total binding energy for the wild-type RBD was reported to be −60.55 kcal/mol, while it was −73.49 kcal/mol for the G446V-RBD complex. This shows that SARS-CoV-2 variants with the G446V mutation increase the binding affinity to enhance the infectivity. Moreover, it can be seen that both the vdW and electrostatic energies are increased in the mutant complex, thus revealing that these findings are consistent with the previous findings reported for other variants (Khan et al., 2020a; Khan et al., 2020d; Khan et al., 2021b; Khan et al., 2021c). The binding free energy results for the RBD–ACE2 complexes are shown in Table 6. Moreover, the results for the NTD were inversed. The total binding energy for the wild-type NTD–mAb complex was −67.77 kcal/mol; −51.25 kcal/mol for the R102S-NTD–mAb complex; while for the G181V-NTD–mAb complex, the total binding energy was −63.68 kcal/mol. Consequently, this implies that these mutations may help the virus to escape the host immune response by weakening the binding of mAb to the NTD of the spike protein, thus evading the neutralization. The binding free energy results for the NTD–mAb complexes are given in Table 6.

**TABLE 6:** Binding free energy calculated as MM/GBSA for each complex. All the energies are given in kcal/mol.

MM/GBSA RBD					
Complex	VDW	ELE	GB	SA	Total binding energy
Wild type	−109.35	−580.14	640.72	−11.78	−60.55
G446V-RBD	−120.35	−590.5	652.31	−14.96	−73.49

MM/GBSA NTD					
Complexes	VDW	ELE	GB	SA	Total binding energy
Wild type	−98.92	−678.15	721.52	−12.22	−67.77
R102S	−94.69	−700.48	755.13	−11.21	−51.25
G181V	−106.51	−478.4	535.69	−14.46	−63.68

## 4 Conclusion

The perpetual appearance of SARS-CoV-2 and its new variants devastated the public health and social fabric around the world. Herein, we connected spike protein’s genomic patterns and phenotypic attributes to understand the evolution, dynamics, and mutational spectrum of SARS-CoV-2 and its new variants circulating in Pakistan. Using the GISAID, a total of 21 unique mutations were identified, and docking with ACE2 and monoclonal antibody (4A8) revealed that mutation G446V in RBD, R102S, and G181V in NTD significantly affect the binding and thus increase the infectivity. The structural-dynamic features of these mutations revealed the global dynamic trend, and the resulting energy calculation further established that the G446V mutation increases the binding affinity toward ACE2, while R102S and G181V help in evading the host immune response. The already available antibodies can be engineered to design potent synthetic antibodies. Moreover, the specified mutant residues in the interface of RBD and ACE2 can be targeted by using *in silico* virtual screening approaches. In conclusion, the current findings revealed the basis for higher infectivity and immune evasion associated with the mutations mentioned previously and structure-based drug discovery against such variants.

## Data Availability

The original contributions presented in the study are included in the article/Supplementary Material; further inquiries can be directed to the corresponding authors.
